# The “Polonium In Vivo” Study: Polonium-210 in Bronchial Lavages of Patients with Suspected Lung Cancer

**DOI:** 10.3390/biomedicines9010004

**Published:** 2020-12-23

**Authors:** Vincenzo Zagà, Maria Sofia Cattaruzza, Paola Martucci, Roberta Pacifici, Rocco Trisolini, Paolo Bartolomei, Raffaela Giacobbe, Marco Patelli, Daniela Paioli, Massimo Esposito, Valeria Fabbri, Silvano Gallus, Giuseppe Gorini

**Affiliations:** 1 Italian Society of Tobaccology SITAB, 40137 Bologna, Italy; vincenzo.zaga@icloud.com; 2 Department of Public Health and Infectious Diseases, Faculty of Medicine and Psychology, La Sapienza University, 00185 Rome, Italy; 3 Bronchial Endoscopy Unit and Interventional Diagnostics-Centre for Tobacco Treatment, AORN A. Cardarelli, 80131 Napoli, Italy; pmartucci2003@yahoo.it (P.M.); raffaela.giacobbe@gmail.com (R.G.); 4 National Observatory on Smoking, Alcohol and Drugs of the National Institute of Health, 00161 Rome, Italy; roberta.pacifici@iss.it; 5 Interventional Pulmonology Unit, Policlinico Universitario A. Gemelli IRCCS, Università Cattolica del Sacro Cuore, 00168 Roma, Italy; rocco.trisolini@policlinicogemelli.it (R.T.); daniela.paioli@policlinicogemelli.it (D.P.); 6 ENEA, 40129 Bologna, Italy; p.bartolomei55@gmail.com; 7 Bronchology Unit, Ospedale Maggiore, 40133 Bologna, Italy; marco.patelli@outlook.it; 8 U-Series Srl, 40128 Bologna, Italy; massimo@u-series.com (M.E.); valeria@u-series.com (V.F.); 9 Department of Environmental Health Sciences, Istituto di Ricerche Farmacologiche Mario Negri IRCCS, 20156 Milan, Italy; silvano.gallus@marionegri.it; 10 Oncologic Network, Prevention and Research Institute (ISPRO), 50139 Florence, Italy; g.gorini@ispro.toscana.it

**Keywords:** polonium-210, lung cancer, smoking status, radon, bronchial lavage

## Abstract

Few studies have reported on polonium-210, a decay breakdown product of radon-222 and lead-210, in human lungs and there has been no study in patients with suspected lung cancer. The main aim of this “Polonium in vivo” study was to evaluate polonium-210 radioactivity in bronchopulmonary systems of smoker, ex-smoker and never smoker patients with suspected lung cancer. Alpha-spectrometric analyses were performed on bronchial lavage (BL) fluids from two Italian hospitals in 2013–2016. Socio-demographic, smoking, occupational and spirometric characteristics, lung cancer confirmation and histologic type and radon-222 concentration in patients’ homes were collected. Seventy BL samples from never (*n =* 13), former (*n =* 35) and current smokers (*n =* 22) were analyzed; polonium-210 was detected in all samples from current and former smokers and in 54% of samples from never smokers (*p* < 0.001; median values: 1.20, 1.43 and 0.40 mBq, respectively). Polonium-210 levels were significantly higher in COPD versus no COPD patients (median value: 3.60 vs. 0.97 mBq; *p* = 0.007); former and current smokers, without and with COPD, had significantly increased polonium-210 levels (*p* = 0.012); 96% of confirmed versus 69% of non-confirmed lung cancer patients recorded detectable polonium-210 levels (*p* = 0.018). A polonium-210 detectable activity was measured in BL samples from all current and former smokers. Polonium-210 in the lungs could be the result of lead-210 entrapment, which, with its half-life of 22 years, could provide a continuous emission of alpha radioactivity, even many years after quitting, thus proposing a possible explanation for the onset of lung cancer, particularly in former smokers.

## 1. Introduction

Tobacco smoke contains human carcinogens, including heavy metals like radioactive lead (^210^Pb; half-life: 22 years), and its daughter isotope polonium (^210^Po; half-life: 138 days); ^210^Pb is a beta-particle emitter, ^210^Po is an alpha-particle emitter (^210^Po), which has been reported to be associated with lung cancer, and in particular it has been hypothesized that it could be implicated in the histotype shift of lung cancer from a squamous cell type to adenocarcinoma [1].

Knowledge about tobacco radioactivity goes back to the sixties, when tobacco companies discovered that polonium was part of tobacco and tobacco smoke [2,3]. Two sources of tobacco radioactivity have been identified: Aitken particles, which are dust particles which contain radioactive radon (^222^Rn) absorbed from the atmosphere, and the uptake from the soil. The Aitken particles deposit and accumulate in the trichome of tobacco leaves where their decay products, ^210^Pb and ^210^Po, form insoluble complexes with resin and are resistant to rain and to tobacco curing. The soil, especially if fertilized with polyphosphates which boost the growth of tobacco plants, contain radium and its decay products. Thus, ^210^Pb and ^210^Po are highly concentrated in tobacco leaves. ^210^Po can be detected both in tobacco smoke gaseous and corpuscular phases, while ^210^Pb only in corpuscular phase [2,3,4].

It has been estimated that a burning cigarette emits into the air about 50% of its radioactive materials and its smoke, inhaled by the smoker, delivers about 32% of the ^210^Po to the lungs. Furthermore, radioactive materials can be inhaled by non-smokers, and are retained in cigarette butts and ashes [5].

In 2011, in the 10 best-selling cigarette brands in Italy, ^210^Pb and ^210^Po average levels were, respectively, 14.6 mBq and 15.8 mBq per cigarette and the annual dose assessment has been estimated to be equal to that from 27–28 chest radiographs (smoking 20 cigarettes/day) [6].

Since the discovery that the presence of radioactivity in tobacco leaves was higher than background levels, tobacco industries tried to remove ^210^Pb and ^210^Po in tobacco through washing tobacco leaves with acid treatments, measuring the radioactivity prior to manufacturing, filtering mainstream smoke, and employing genetic engineering techniques in order to reduce radioactivity in the leaves. As the results were not satisfactory or could compromise the optimal absorption of nicotine into the brain, they decided to stop research in this area and eliminate any evidence that might put their companies at risk for smoking and health litigation [5,7].

Indeed, since high concentrations of polonium have been found in bronchial epithelium of smokers, it was hypothesized that polonium may be implicated in the initiation of lung cancer [5,8]. Actually, ^210^Pb and ^210^Po might be implicated also in the initiation of other tumors, since the radioactive particles from the bronchopulmonary system might spread throughout the body and reach various organs and tissues. In smokers compared to non-smokers, ^210^Po levels are significantly higher in the blood, urine, liver, kidney, heart and skeletal muscles [3,9,10].

Although research indicating polonium as one of the major tobacco carcinogens is encouraging, a recent systematic review of this issue has shown that the study of radioactivity of tobacco smoke has been scientifically stagnant for 40 years. On the other hand, the extent to which the tobacco industry has understood the risks of polonium and has discussed its implications is impressive [11,12].

To our knowledge, few studies have reported measurements of ^210^Po in human lungs, and this is the first study to report levels of ^210^Po in smoker, ex-smoker and non-smoker patients with lung cancer.

The main aim of this paper was to show results of the “Polonium in vivo” study, aimed at evaluating ^210^Po radioactivity in bronchopulmonary systems among patients with suspected and confirmed lung cancer.

## 2. Materials and Methods

All consecutive patients undergoing a bronchoscopy for suspected lung cancer in the pneumology divisions of two major hospitals in Northern (Ospedale Maggiore in Bologna) and Southern Italy (Ospedale Cardarelli, Naples) were admitted if they were aged 35–85 years.

Patients were enrolled into three groups according to their smoking status: “never smokers”, if they had smoked < 100 cigarettes in their lifetime and were not exposed to second-hand smoke in the past 15 years; “former smokers”, if they had quit at least 5 years ago, with at least 20 years of smoking duration; “current smokers”, if they had smoked in the last 20 years.

The study was conducted between January 2013 and September 2014 and it was reopened in the first half of 2016 to increase the enrolment in the first group of “never smokers” patients; thus, 6 additional never smoking patients with suspected lung cancer were added to the study. Assuming a 50% difference in chronic obstructive pulmonary disease (COPD) prevalence between ever and never smokers with lung cancer [13], a sample size of 18 patients per each smoking status group was necessary to conduct the study, with a significant level α = 0.05 and a 0.8 power.

The study protocol was approved by the Bologna Local Health Unit Ethical Committee on December 2012 (Prot. N. 1539/CE).

An information letter and the related consent form were provided to patients. An information letter was also sent to their general practitioner.

Alpha spectrometric analyses were carried on bronchial lavage (BL) fluids collected from recruited patients during bronchoscopy procedures; 15 mL of normal saline solution was used during the procedure. Fluids were collected into sterilized test tubes from the bronchoscopy and were sent to the laboratory.

Sociodemographic variables (gender, age), smoking history (pack-year of smoking, years since quitting), past occupational exposure to lung carcinogens and two spirometry measures were collected: forced expiratory volume in the first second (FEV1), and the FEV1/FVC ratio (Tiffeneau index), i.e., the proportion of FEV1 to the full forced vital capacity (FVC). According to the GOLD classification, patients were classified as having or not COPD, and, among COPD patients, COPD severity level [14]. Results of cytological examinations of BL cells were collected: non-confirmed lung cancer or lung cancer histologic types (adenocarcinoma, squamous cell carcinoma, other non-small cell lung cancer (NSCLC), and small cell lung cancer (SCLC)).

Since ^210^Po is a decay product of radon 222 (^222^Rn), ^222^Rn concentration at the patients’ homes was also measured.

In summary, BL fluids from 70 patients were collected and analyzed (Table S1 in Supplementary Materials); for 34 and 11 patients, respectively, radon and spirometric measures were not available and, for 10 patients only gender and smoking status were available.

Sample preparation and ^210^Po analyses were carried out according to standard procedures [15]. Samples were spiked with radioactive tracer ^209^Po at known concentrations. Sample decomposition was performed using conventional digestion techniques with acids. Then, samples were loaded in an extraction chromatographic resin (Sr Resin, Triskem International) to separate lead and polonium and to obtain a solution containing polonium that was recovered in low-molarity hydrochloric acid and then injected in a cell with a silver disc for ^210^Po deposition. The silver disc with deposited ^210^Po was then measured using alpha spectrometry with silicon detectors in a vacuum chamber and a multichannel analyzer. ^210^Po results referred to the date of laboratory analysis; correction to the sampling date was not needed because it is assumed that in tobacco smoke the ^210^Po/^210^Pb activity ratio is 1 [6]. If ^210^Po activity was below the detection limit, it was assumed to be zero.

Radon (^222^Rn) measurements were carried out by CR-39 nuclear track detectors. These passive detectors were installed in the patients’ homes for about three months. Radon particles in air damage the sensor, producing tracks that were analyzed in U-Series laboratory according to ISO 11665-4:2012 [16,17].

In order to test differences in categorical and continuous variables by smoking status, Chi-square test and Student’s t-test were used, respectively. The Kruskal–Wallis and the Mann–Whitney two-sample statistics were used for testing differences in 210Po levels by smoking status, and by having or not COPD or lung cancer.

## 3. Results

A total of 70 patients with suspected lung cancer were recruited and for 47 of them lung cancer confirmation was obtained during the collection of data. Table 1 reports the main characteristics of patients participating in the “Polonium in vivo” study.

Among 60 patients with complete data, except for spirometric and radon measures, 75% were men and the average age was 62 years among current smokers and 71 years in never and former smokers (*p* = 0.017). The cumulative exposure to tobacco smoke was significantly lower in former compared to current smokers (43 vs. 64 pack-years on average, respectively; *p* = 0.012), and former smokers had stopped a mean of 17 years ago. Past occupational exposure to lung carcinogens was recorded in 27% of patients, with no significant differences by smoking status. Lung cancer was diagnosed in 50% of never, 82% of former, and 88% of current smokers; adenocarcinoma was the most frequent histopathological type among never and former smokers (60%), whereas among current smokers it was squamous cell carcinoma (53%). Among 49 patients who also had complete data for spirometric measures, FEV1 was significantly higher in never compared to former and current smokers (103, 87 and 69 on average, respectively; *p* = 0.004). COPD was detected in 8 former smokers (2 mild and 6 moderate COPD), and in 4 current smokers (2 moderate and 2 severe COPD). A total of 11 out of 12 COPD patients (92%) had confirmed lung cancer.

Table 2 reports levels of ^210^Po in patients with suspected lung cancer. Considering all 70 patients with ^210^Po measurements, ^210^Po was detected in 100% of BL fluids from current or former smokers and in 54% from never smokers (i.e., ^210^P > 0 mBq) (*p* < 0.001; Table 2).

We did not observe any statistically significant difference in ^210^Po levels between current and former smokers (Table 2). For two never smokers, ^210^Po levels were very high: 16.35 mBq and 16.66 mBq. These two subjects lived in houses with the highest ^222^Rn concentration values recorded in this study: 330 Bq/m^3^ and 1100 Bq/m^3^, respectively. Excluding these two never smokers, the ^210^Po median value among never smokers was 0.00 mBq (interquartile range: 0.00–1.50 mBq) and the differences among ever versus never smokers significantly increased (Kruskal–Wallis test *p*-value = 0.013).

**Figure 1:**
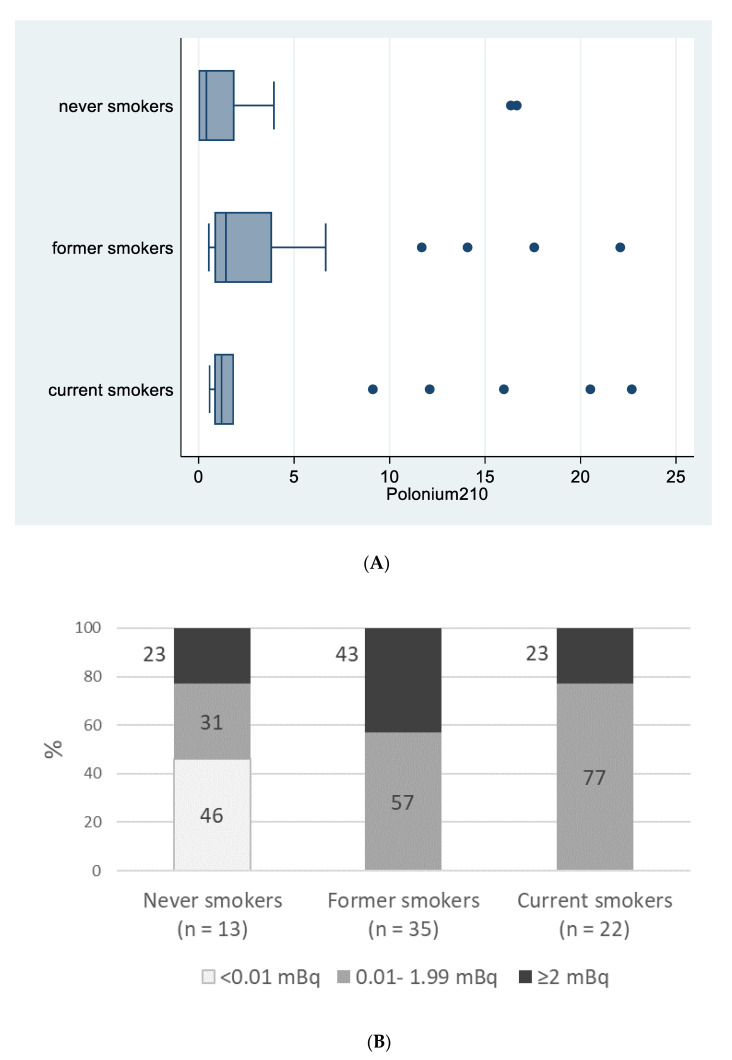
Polonium-210 (in mBq) by smoking status, “Polonium in vivo” study (*n =* 70). (**A**) Box plot of polonium-210 (in mBq) by smoking status. The left and right borders of the box are the lower and upper quartiles; the solid line in the box is the median. The “whiskers”, that is, the lower and upper adjacent values, are used for identifying extreme values in the tails of the distribution. Dots greater than the upper adjacent value, represent outliers. (**B**) Polonium-210 levels (<0.01 mBq; 0.01–1.99 mBq; >=2 mBq) by smoking status.

Figure 1A reports ^210^Po median values for never, former and current smokers (0.41, 1.43, 1.20, mBq, respectively; *p* = 0.149) and Figure 1B reports percentages of patients in the three ^210^Po categories (0, <2 and ≥2 mBq) according to their smoking status (*p* < 0.001).

**Figure 2:**
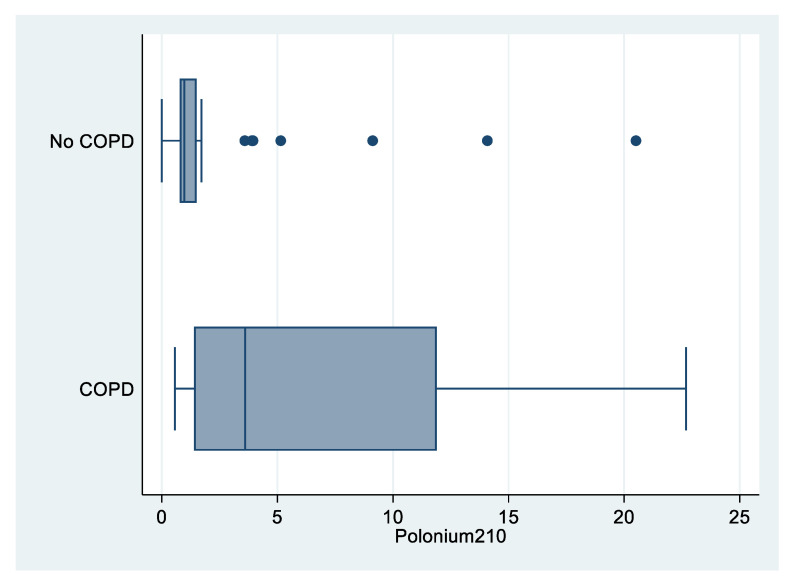
Box plot of polonium-210 (in mBq) by COPD status, “Polonium in vivo” study (*n =* 49; 21 missing; COPD *n =* 12; no COPD *n =* 37). The left and right borders of the box are the lower and upper quartiles; the solid line in the box is the median. The “whiskers”, that is, the lower and upper adjacent values are used for identifying extreme values in the tails of the distribution. Dots greater than the upper adjacent value, represent outliers.

As shown in Figure 2, levels of ^210^Po were higher among COPD patients compared to patients with no COPD (median value: 3.60 vs. 0.97 mBq; *p* = 0.007; Table 2).

**Figure 3:**
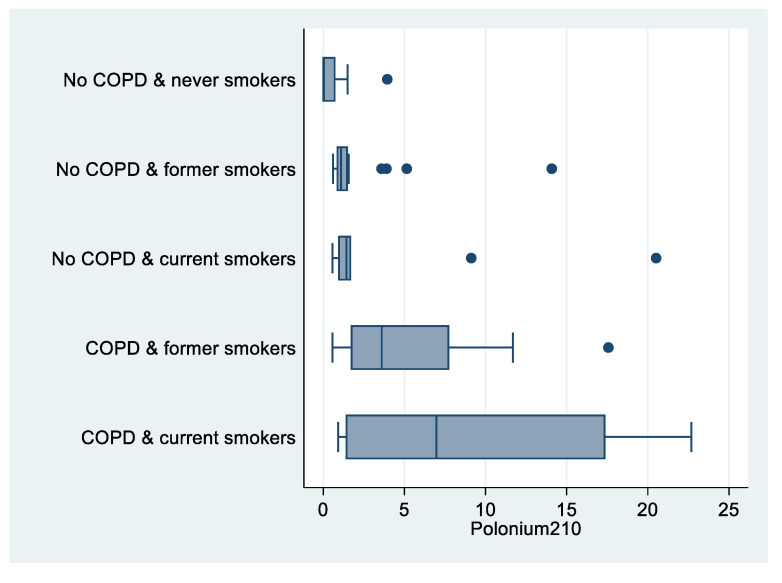
Box plot of polonium-210 (in mBq) by COPD and smoking status, “Polonium in vivo” study (*n =* 49; 21 missing). The left and right borders of the box are the lower and upper quartiles; the solid line in the box is the median. The “whiskers”, that is, the lower and upper adjacent values, are used for identifying extreme values in the tails of the distribution. Dots greater than the upper adjacent value represent outliers.

There were significant differences in ^210^Po levels among never smokers with no COPD (6 out of 8 ^210^Po measures below the detection limit), compared to current smokers with COPD (median value: 6.97 mBq; *p* = 0.012; Table 2, Figure 3).

**Figure 4:**
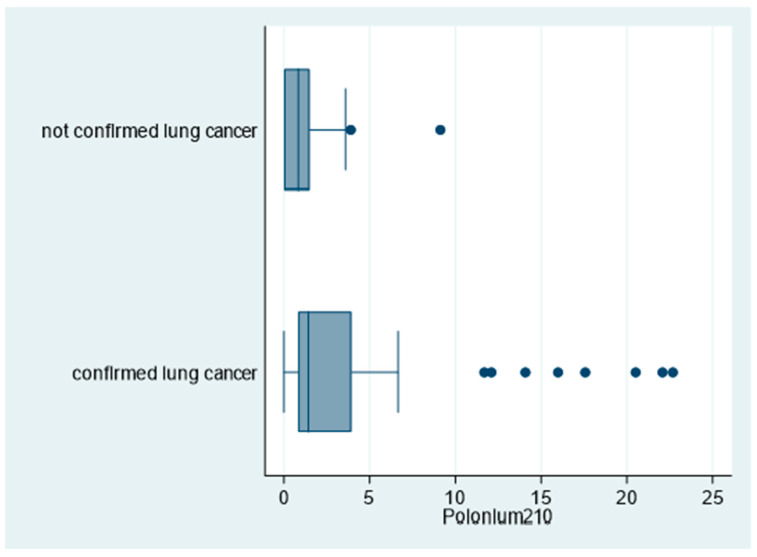
Box plot of polonium-210 (in mBq) by lung cancer status, “Polonium in vivo” study (*n =* 60; 10 missing; not confirmed lung cancer *n =* 13; confirmed lung cancer *n =* 47). The left and right borders of the box are the lower and upper quartiles; the solid line in the box is the median. The “whiskers”, that is, the lower and upper adjacent values, are used for identifying extreme values in the tails of the distribution. Dots greater than the upper adjacent value, represent outliers.

Moreover, as shown in Figure 4, ^210^Po levels were significantly higher among patients with confirmed lung cancer compared to patients with no lung cancer confirmation (96% and 69%, respectively for ^210^P > 0 mBq; *p* = 0.018; Table 2).

No differences in ^210^Po levels were observed according to past occupational exposure to lung carcinogens or by histopathological type of lung cancer (data not shown).

A total of 34 out of 36 patients with radon measurements recorded radon levels ≤300 Bq/m^3^, with ^210^Po median value = 1.20 mBq. Only two patients recorded radon concentrations >300 Bq/m^3^, with higher ^210^Po levels (median value = 16.50 mBq; *p* = 0.027; Table 2). There were no differences in radon concentration by smoking status, excluding the two never smokers with very high radon concentration at home.

Considering only 47 patients with confirmed lung cancer, differences in ^210^Po levels by smoking status did not change significantly (Table 2).

## 4. Discussion

To our knowledge, this is the first study measuring ^210^Po levels from BL fluids in patients with suspected or confirmed lung cancer, overall and by smoking status. Former and current smokers had significantly higher levels of ^210^Po compared to never smokers. Moreover, there were significant differences in ^210^Po levels among never smokers with no COPD compared to current smokers with COPD, and patients with confirmed lung cancer, compared to those with non-confirmed lung cancer, recorded significantly higher ^210^Po levels.

The finding of polonium in BL provides hints for etiological hypotheses. Polonium is an alpha decay breakdown product of radon and a beta decay breakdown product of radioactive lead isotopes, all of which can be associated with lung cancer development. Inhaled tobacco smoke compounds are readily dispersed and cleaned through the bronchial mucociliary clearance (MCC) [2,3]. High ^210^Po levels in smokers’ lungs could be due to the accumulation of insoluble ^210^Pb particles, the father of ^210^Po, due to an increasingly progressive MCC impairment in smokers, reaching a minimum in COPD patients. In all heavy smokers with COPD there are metaplastic lesions of the ciliated epithelium [18]. Radionuclides thus can better penetrate in de-epithelialized or poorly ciliated areas where mucus stagnates more [3,19]. Due to the relatively long half-life of ^210^Pb (22 years) and the progressive MCC impairment, particularly in COPD patients, the entrapment of ^210^Pb and ^210^Po can lead to replenishment of alpha radioactivity with persistent lung cancer risk even after many years. Thus, this study provides an etiological hypothesis for those studies which had identified COPD as an independent risk factor for lung cancer [20,21,22].

Practical consequences of this hypothesis are: (i) identifying COPD patients as early as possible; (ii) administering pharmacological treatments to improve MCC and to remove ^210^Pb particles.

The World Health Organization recommends a reference level of 100 Bq/m^3^ for ^222^Rn concentration in homes and the International Commission for Radiological Protection has also recommended a level not exceeding 300 Bq/m^3^ [23,24]; ^222^Rn concentration in homes >300 Bq/m^3^ may be the cause of ^210^Po detection in never smokers, which happened in this study. Further studies are needed to confirm this finding.

Adenocarcinoma was the most common histological type of cancer among former smokers in this study; part of these cancer cases could be induced by ^210^Pb. Animal studies confirm this hypothesis [25,26].

This study has some limitations and some strengths. One limitation is that the number of never smoker patients was lower than that estimated in the sample size calculation (18 patients), since never smokers with suspected lung cancer were very few. Moreover, for some patients not all data were available, since it was not possible for all recruited patients to collect radon measurements at their homes and to administer spirometry tests. The cross-sectional nature of the study does not allow us to evaluate a cause–effect relationship, but this is, to our knowledge, the first study that has found an association between radioactivity (^210^Po) in the bronchopulmonary systems of patients and lung cancer. We could not compare ^210^Po measurements between patients and healthy controls for ethical reasons due to the invasive procedure of BL. Further studies could consider evaluating ^210^Po measurements in all patients with indication of BL (not only for suspected lung cancer).

The strength of this study is that results showed ^210^Po levels in the lungs of patients with lung cancer. Moreover, ^210^Po was detected in 100% of BL fluids from current and former smokers (who had quit smoking for at least 5 years) and in 54% of never smokers (*p* < 0.001). Lastly, former smokers had levels of ^210^Po similar to those of current smokers and significantly higher levels than never smokers.

## 5. Conclusions

In former smokers, the presence of detectable 2^10^Po may be supported by its “father” ^210^Pb, due to reduced MCC. This mechanism could partially explain the onset of lung cancer in former smokers even many years after quitting. If confirmed by large longitudinal studies, our study may have important implications from a public health perspective. Pharmacological treatments improving MCC might in fact reduce ^210^Pb and ^210^Po levels in the lungs, thus substantially decreasing the risk of lung cancer in current smokers and particularly in former smokers.

## Figures and Tables

**Table 1:** Characteristics of patients participating in the “Polonium in vivo” study by smoking status (*n =* 70).

Variables and Characteristics		Never Smokers	Former Smoker	Current Smokers	*p*-Value
Gender, *n* (%)	Men	5 (38.5)	27 (77)	20 (90.9)	0.002 *
	Women	8 (61.5)	8 (22.9)	2 (9.1)	
Hospitals, *n* (%)	Bologna	2 (20.0)	24 (72.7)	5 (29.4)	0.002 *
	Naples	8 (80.0)	9 (27.3)	12 (70.6)	
	Missing	3	2	5	
Age, years	Mean (SD)	71.5 (11.4)	70.9 (8.7)	62.3 (11.0)	0.017 ^§^
	Missing	3	2	5	
Pack-year ^#^	Mean (SD)	0.0 (0.0)	42.9 (24.8)	63.6 (29.8)	0.012 ^§^
	Missing	3	2	5	
Quit since, years	Mean (SD)	-	17.4 (10.3)	-	
	Missing	-	2	-	
Past occupational exposure to lung	No	9 (90.0)	24 (72.7)	11 (64.7)	0.355 *
carcinogens, *n* (%)	Yes	1 (10.0)	9 (27.3)	6 (35.3)	
	Missing	3	2	5	
FEV1/FVC ratio, %	Mean (SD)	86.1 (18.0)	77.4 (16.8)	77.6 (21.3)	0.221 ^§^
	Missing	5	7	9	
FEV1, %	Mean (SD)	103 (18.7)	86.8 (24.2)	68.5 (20.8)	0.004 ^§^
	Missing	5	7	9	
COPD, GOLD classification	No	8 (100.0)	20 (71.4)	9 (69.2)	0.210 *
	Yes	0 (0.0)	8 (28.6)	4 (30.8)	
	Missing	5	7	9	
Lung cancer, *n* (%)	No	5 (50.0)	6 (18.2)	2 (11.8)	0.051 *
	Yes	5 (50.0)	27 (81.8)	15 (88.2)	
	Missing	3	2	5	
Histology, *n* (%)	Adenocarcinoma	3 (30.0)	16 (48.5)	4 (23.5)	
	Squamous cell carcinoma	1 (10.0)	7 (21.2)	8 (47.1)	
	Other NSCLC	1 (10.0)	2 (6.1)	2 (11.8)	0.159 *
	SCLC	0 (0.0)	2 (6.1)	1 (5.9)	
	No lung cancer	5 (50.0)	6 (18.2)	2 (11.8)	
	Missing	3	2	5	
Overall, *n* (%)		13	35	22	

* *p*-values from Chi-square test; ^§^ *p*-values from Student’s t-test. ^#^ pack-year = a way to measure the amount a person has smoked over a long period of time. It is calculated by multiplying the number of packs of cigarettes smoked per day by the number of years the person has smoked.

**Table 2:** Levels of ^210^Po in 70 patients with suspected lung cancer from the “Polonium in vivo” study by smoking status and by having or not COPD or lung cancer (if ^210^Po activity was below the detection limit it was assumed to be zero).

Variables and Characteristics		*n*	^210^Po [mBq] Median (IQR^)	*p*-Value ^§^	0 mBq *n* (%)	0.01–1.99 mBq *n* (%)	≥2 mBq *n* (%)	*p*-Value *
Smoking status	Never smoker	13	0.41 (0.00–1.88)		6 (46.2)	4 (30.8)	3 (23.1)	
	Former smokers	35	1.43 (0.83–3.85)	0.149	0 (0.0)	20 (57.1)	15 (42.9)	<0.001
	Current smokers	22	1.20 (0.82–1.85)		0 (0.0)	17 (77.3)	5 (22.7)	
COPD	No	37	0.97 (0.78–1.50)		6 (16.2)	24 (64.9)	7 (18.9)	
	Yes	12	3.60 (1.40–11.89)	0.007	0 (0.0)	4 (33.3)	8 (66.7)	0.006
	Missing	21	-		0	13	8	
COPD and smoking	No COPD and never smokers	8	0.00 (0.00–0.75)		6 (75.0)	1 (12.5)	1 (12.5)	
status	No COPD and former smokers	20	1.09 (0.83–1.52)		0 (0.0)	16 (80.0)	4 (20.0)	
	No COPD and current smokers	9	1.43 (0.92–1.72)	0.012	0 (0.0)	7 (77.8)	2 (22.2)	<0.001
	COPD and former smokers	8	3.60 (1.69–7.76)		0 (0.0)	2 (25.0)	6 (75.0)	
	COPD and current smokers	4	6.97 (1.39–17.39)		0 (0.0)	2 (50.0)	2 (50.0)	
	Missing	21	-		0	13	8	
Lung cancer	No	13	0.83 (0.00–1.50)		4 (30.8)	6 (46.2)	3 (23.1)	
	Yes	47	1.43 (0.82–3.94)	0.071	2 (4.3)	28 (59.6)	17 (36.2)	0.018
	Missing	10	-		0	7	3	
Radon	≤300	34	1.20 (0.78–1.50)		4 (11.8)	23 (67.7)	7 (20.6)	
(Bq/m^3^)	>300	2	16.50 (16.35–16.66)	0.027	0 (0.0)	0 (0.0)	2 (100.0)	0.042
	Missing	34	-		2	18	14	

^ IQR: 1st and 3rd interquartile range; ^§^ *p*-values from the Kruskal–Wallis tests (for >2 medians) or the Mann–Whitney two-sample statistic (for 2 medians); * *p*-values from Chi-square test.

## Data Availability

The data presented in this study are available in Table S1 in Supplementary Materials.

## Supplementary Materials

The following are available online at https://www.mdpi.com/2227-9059/9/1/4/s1, Table S1: Summary data.
