# Supplementary material for: The “Polonium In Vivo” Study: Polonium-210 in Bronchial Lavages of Patients with Suspected Lung Cancer
Source: Biomedicines. 2020 Dec 23;9(1):4. doi: 10.3390/biomedicines9010004 (PMC7822435; doi:10.3390/biomedicines9010004)
Supplement: Supplementary file 1 [file biomedicines-09-00004-s001.pdf]

biomedicines

MDPI

Supplementary Materials

Table S1. Summary data.

ID	Smoking Status	Gender	Age	Pack-year	Lung Cancer *	Bpco	Occ. Exp.	^{210}Po (mBq)$^{++}$			^{222}Rn (Bq/m^3)**		
1	Current	M	59	64	S	yes	no	22.68	±	4.91	15	±	6
2	Current	M	71	96	S	no	no	0.78	±	0.18	-	-	-
3	Current	M	73	30	A	-	yes	0.82	±	0.13	-	-	-
4	Current	M	73	105	S	no	no	1.43	±	0.14	27	±	7
5	Current	M	80	97	S	no	no	20.51	±	1.62	-	-	-
6	Current	M	46	60	S	no	no	0.57	±	0.06	28	±	7
7	Current	M	42	30	S	-	no	15.98	±	2.89	-	-	-
8	Current	M	62	50	no	no	no	9.12	±	0.60	57	±	12
9	Current	M	72	90	S	yes	yes	12.10	±	0.60	-	-	-
10	Current	M	51	22	other	no	yes	1.72	±	0.20	-	-	-
11	Current	M	65	48	S	yes	no	1.85	±	0.28	-	-	-
12	Current	M	72	99	M	no	yes	1.15	±	0.20	21	±	9
13	Current	M	46	99	other	no	yes	0.92	±	0.12	220	±	30
14	Current	M	64	35	A	-	no	0.81	±	0.13	-	-	-
15	Current	F	63	40	A	yes	no	0.93	±	0.11	-	-	-
16	Current	M	64	84	no	no	yes	1.43	±	0.18	93^{+}	-	-
17	Current	F	56	33	A	-	no	1.31	±	0.11	-	-	-
18	Current	M	-	-	-	-	-	1.21	±	0.12	64	±	13
19	Current	M	-	-	-	-	-	1.00	±	0.14	32	±	5
20	Current	M	-	-	-	-	-	1.20	±	0.22	90	±	14
21	Current	M	-	-	-	-	-	0.75	±	0.12	-	-	-
22	Current	M	-	-	-	-	-	0.73	±	0.12	140^{+}	-	-
23	Former	M	60	25	S	no	yes	0.78	±	0.11	25	±	7
24	Former	M	65	80	A	no	yes	1.42	±	0.18	98	±	17
25	Former	F	56	46	S	no	no	0.91	±	0.13	44	±	9
26	Former	M	78	75	S	no	no	0.82	±	0.09	39	±	9
27	Former	M	63	49	S	yes	no	3.66	±	0.31	-	-	-
28	Former	F	80	20	A	no	no	0.86	±	0.10	16	-	-
29	Former	M	82	52	S	no	no	5.14	±	0.45	-	-	-
30	Former	M	69	48	M	-	no	6.65	±	0.95	-	-	-
31	Former	M	62	45	other	-	no	22.08	±	1.18	-	-	-
32	Former	M	68	10	A	no	yes	1.43	±	0.15	66	±	14
33	Former	M	64	50	no	no	yes	0.78	±	0.17	47	±	11
34	Former	F	77	48	no	yes	no	0.57	±	0.12	-	-	-
35	Former	M	68	40	A	no	no	1.57	±	0.23	83	±	16
36	Former	M	73	8	S	no	no	0.72	±	0.09	57	±	14
37	Former	F	69	41	no	no	no	1.21	±	0.20	140	±	20
38	Former	M	80	45	A	no	yes	0.60	±	0.10	-	-	-
39	Former	M	84	30	A	no	yes	0.83	±	0.13	-	-	-
40	Former	F	50	14	no	no	no	3.89	±	0.26	-	-	-
41	Former	M	70	18	A	no	no	1.27	±	0.15	150^{+}	-	-
42	Former	M	83	49	A	no	no	0.97	±	0.16	-	-	-
43	Former	M	72	60	A	-	no	0.53	±	0.09	-	-	-
44	Former	M	79	36	A	yes	no	3.85	±	0.21	-	-	-
45	Former	M	77	37	S	yes	no	2.43	±	0.44	62	±	15
46	Former	M	69	126	A	yes	no	17.57	±	0.60	-	-	-
47	Former	F	63	10	A	yes	no	3.55	±	0.21	65	±	15
48	Former	M	82	55	no	no	no	3.59	±	0.29	-	-	-
49	Former	F	57	11	A	-	no	4.16	±	0.30	57	±	15
50	Former	M	82	25	no	no	yes	0.83	±	0.12	65	±	14

51	Former	F	65	36	other	no	no	14.08	±	2.64	-	-	-
52	Former	M	76	40	A	-	no	3.50	±	0.32	80	±	15
53	Former	M	69	45	A	no	yes	1.47	±	0.66	60	±	15
54	Former	M	72	90	M	yes	no	11.68	±	1.53	-	-	-
55	Former	M	77	53	A	yes	yes	0.95	±	0.21	-	-	-
56	Former	M	-	-	-	-	-	0.90	±	0.09	-	-	-
57	Former	M	-	-	-	-	-	3.30	±	0.60	-	-	-
58	Never	F	74	0	no	no	no	1.50	±	0.16	69	±	14
59	Never	F	72	0	S	-	no	1.88	±	0.27	-		
60	Never	F	67	0	A	no	no	3.94	±	0.29	99	±	14
61	Never	M	85	0	A	-	yes	0.41	±	0.07	-	-	-
62	Never	F	74	0	no	no	no	<1.8		-	-	-	-
63	Never	M	52	0	A	no	no	<0.9		-	-	-	-
64	Never	M	69	0	no	no	no	<0.60		-	210+	-	-
65	Never	M	71	0	other	no	no	<0.40		-	121+	-	-
66	Never	F	80	0	no	no	no	<1.4		-	170+	-	-
67	Never	F	48	0	no	no	no	<1.31		-	83+	-	-
68	Never	F	-	0	-	-	-	0.96	±	0.18	-	-	-
69	Never	M	-	0	-	-	-	16.35	±	4.0	330+	-	-
70	Never	F	-	0	-	-	-	16.66	±	6.0	1100+	-	-

* Lung Cancer: S = Squamous cell carcinoma; A = Adenocarcinoma; other = Other non-small cell carcinoma; M = Microcitoma; no = non-confirmed lung cancer; [++] 1 sigma combined uncertainty; [**] Extended uncertainty (cover factor k = 2); [+] Qualitative value.
